# Age-stratified gradients of colorectal neoplasia and an exploratory endoscopic red-flag composite in a lesion-confirmed screening colonoscopy cohort

**DOI:** 10.3389/fonc.2026.1842525

**Published:** 2026-10-02

**Authors:** Lina Du, Jingzhi Su, Jihua Dai, Peipei Pu, Li Meng, Feiyan Zhao, Lili Pang, Sizhu Li, Yanxia Zhang, Xin Rong, Huihong Zhai, Sufang Shen

**Affiliations:** 1 Department of Gastroenterology, Beijing Genertec Aerospace Hospital, Beijing, China; 2 Department of Health Management, Beijing Genertec Aerospace Hospital, Beijing, China; 3 Department of Gastroenterology, Beijing Renhe Hospital, Beijing, China; 4 Department of Internal Medicine, Beijing Tongzhou District Maternal and Child Health Care Hospital, Beijing, China; 5 Department of General Practice, Beijing Daxing District Jiugong Hospital, Beijing, China; 6 Department of Gastroenterology, Xuanwu Hospital, Capital Medical University, Beijing, China

**Keywords:** age stratification, colonoscopy, colorectal neoplasia, exploratory analysis, *Helicobacter pylori*, red-flag composite

## Abstract

**9Objective:**

To characterise age-related gradients in colorectal lesion severity and prespecified high-risk endoscopic features in a lesion-confirmed screening colonoscopy cohort and to evaluate an investigator-defined red-flag composite as an exploratory marker of advanced pathology. *Helicobacter pylori* was assessed as a secondary exposure.

**Methods:**

This retrospective cohort included 291 participants with histologically confirmed lesions in four age groups (36–45, 46–55, 56–65 and 66–72 years). Logistic regression evaluated predictors of advanced neoplasia (high-grade dysplasia [HGD] or adenocarcinoma); adenocarcinoma-only and age × *H. pylori* interaction analyses were exploratory because events were sparse.

**Results:**

With advancing age, body mass index, *H. pylori* positivity, adenoma, sessile serrated lesions, HGD, adenocarcinoma and high-risk endoscopic features increased, whereas hyperplastic polyps decreased (all P < 0.05). Age (per 10 years), male sex, *H. pylori* positivity, lesion size ≥10 mm, proximal location and JNET 2B/3 pattern were independently associated with advanced neoplasia. Red-flag positivity (≥2 of four features) increased from 5.2% to 32.7% and enriched for adenocarcinoma, although positive predictive values were imprecise and non-monotonic.

**Conclusions:**

Colorectal lesion severity and high-risk endoscopic features showed an age-related gradient. The red-flag composite is an exploratory descriptive construct, not a validated predictive score. *H. pylori* findings, especially interaction analyses, are hypothesis-generating and require prospective validation.

## 1 Introduction

Colorectal neoplasia encompasses adenomas, sessile serrated lesions (SSLs), high-grade intraepithelial neoplasia and adenocarcinoma, and it remains a major global cancer burden. GLOBOCAN 2020 estimated more than 1.9 million new colorectal cancer (CRC) cases and approximately 935,000 deaths worldwide, placing CRC among the most commonly diagnosed malignancies and leading causes of cancer mortality (1). Contemporary reviews and guidelines emphasise that colorectal carcinogenesis is heterogeneous, involves both conventional adenoma–carcinoma and serrated pathways and can be substantially mitigated by effective screening and removal of precursor lesions (2, 3). Despite the proven value of colonoscopy-based prevention, participation and resource availability remain variable, and pragmatic risk-stratification approaches are still needed to help prioritise lesions and patients who may require closer evaluation (3).

Age is a key unmodifiable determinant of colorectal neoplasia, but the relationship is better characterised as a progressive, heterogeneous age-associated gradient rather than a single fixed percentage increase across all populations. A recent meta-analysis of colorectal polyp distribution reported age-related differences in polyp location and burden (4), while large colonoscopy datasets have shown that adenoma detection and advanced neoplasia generally increase across successive decades after age 50 (5). Focused clinical series have similarly highlighted that adenoma detection varies by age and sex, supporting age-aware interpretation of colonoscopy findings (6). Mechanistically, ageing-associated genomic instability, epigenetic drift, clonal selection, chronic low-grade inflammation and declining immune surveillance can facilitate accumulation of alterations in pathways such as *APC*, *KRAS*, *TP53*, mismatch repair and serrated-pathway signalling (7). These observations support detailed age-stratified analyses while also cautioning against overgeneralised numerical claims about age effects.

*Helicobacter pylori* infection is an established class I gastric carcinogen and has also been reported in observational studies to have modest associations with colorectal adenoma, advanced adenoma and CRC (8, 9). However, the evidence remains heterogeneous; residual confounding, differences in *H. pylori* testing methods, eradication history and population background risk limit causal interpretation, and Mendelian randomisation analyses have not consistently supported a causal effect on CRC risk (10). Proposed biological links include systemic inflammation, hypergastrinaemia, immune modulation and microbiome alterations, but these pathways have not been directly measured in the present cohort (8, 11). Therefore, *H. pylori* status has been evaluated as a secondary exploratory exposure rather than as a central determinant of colorectal carcinogenesis.

Accordingly, using a retrospective lesion-confirmed screening colonoscopy cohort, we provide a granular, age-stratified characterisation across four prespecified age groups of (i) the colorectal lesion spectrum and (ii) prespecified endoscopic features linked to advanced histology. We further evaluate an investigator-defined, pragmatic red-flag composite as an exploratory triage signal for lesions enriched for advanced pathology, with descriptive sensitivity analyses of alternative cutoffs. Finally, we assess *H. pylori* status and age × *H. pylori* effect modification only as hypothesis-generating analyses because adenocarcinoma events were sparse. Collectively, this work aims to provide an age-aware descriptive framework and to motivate prospective, multicentre validation, rather than to propose a validated scoring system or change current screening algorithms.

## 2 Materials and methods

### 2.1 Study design and population

This single-centre, retrospective cohort study consecutively enrolled participants who underwent colonoscopy screening at the Endoscopy Centre of our hospital between 1 January 2020 and 31 December 2024 and had at least 1 histologically confirmed colorectal lesion. The study protocol was approved by the Ethics Committee of China Aerospace Science & Industry Corporation 731 Hospital (approval no. 2017-P2-147-02), and all participants provided written informed consent for colonoscopic examination and data use. Because the cohort was restricted to participants with detected, histologically confirmed lesions, lesion proportions were interpreted as distributions within a lesion-confirmed screening cohort rather than as population prevalence among all individuals undergoing screening.

### 2.2 Colonoscopy screening

All examinations were performed by 3 senior endoscopists, each with ≥8 years of experience, using Olympus CF-HQ290 and EVIS X1 systems (Olympus Corporation, Tokyo, Japan). Standardised bowel preparation consisted of 3 L of polyethylene glycol electrolyte solution taken in split doses. Complete colonoscopy to the caecum was required, with a withdrawal time of ≥6 min for mucosal inspection, excluding time spent on biopsy or treatment. Lesions were classified according to the Paris endoscopic classification (12) and Japan Narrow-Band Imaging (NBI) Expert Team (JNET) classification under magnifying NBI (13).

For each lesion recorded, the following were noted: location, categorised as proximal (caecum to splenic flexure) or distal (splenic flexure to anal verge); largest diameter, measured using the 7 mm open biopsy forceps as an internal reference; and surface structural disruption. Surface structural disruption was defined as visible fissuring or ulcer-like breakage of the mucosal surface, destroyed or interrupted pit/groove architecture, amorphous or irregular microvascular pattern, or focal loss of an otherwise recognisable mucosal micro-pattern under white-light chromoendoscopy and/or NBI, after washing and suctioning to exclude mucus, stool, bubbles or light-reflection artefacts. Representative images were stored in duplicate and independently reviewed by 2 senior endoscopists, with any disagreements resolved by consensus.

### 2.3 Histopathological processing and diagnosis

Biopsy or resection specimens were fixed in 4% neutral-buffered formalin, serially sectioned (≥6 levels), and stained with haematoxylin and eosin. Two gastrointestinal pathologists, blinded to age and *H. pylori* status, independently reviewed each specimen. Diagnoses were made according to the 2019 WHO Classification of Tumours: Digestive System Tumours (14). When multiple lesions were present, the most advanced histological lesion per participant was used for participant-level analyses.

### 2.4 ^13^C-urea breath test

After an overnight fast of ≥8 h, participants exhaled into a baseline collection bag and then ingested a 75 mg ^13^C-urea solution containing citric acid. A second breath sample was obtained 30 min later; δ^13^C enrichment was measured by isotope-ratio mass spectrometry (ABCA2; Sercon Ltd., Crewe, UK) and *H. pylori* positivity was defined as a delta value of ≥3.5‰.

### 2.5 Bowel-preparation quality

The Boston Bowel Preparation Scale was applied: each of the right, transverse and left colon segments was scored 0–3, yielding a total score of 0–9 (15). Mean total scores were compared across age strata and included as a quality-control descriptor.

### 2.6 Red-flag feature composite

A pragmatic ‘red-flag’ composite was prespecified as the presence of ≥2 of 4 higher-specificity endoscopic features frequently associated with advanced histology and/or invasion risk in routine practice: lesion size ≥10 mm (16); proximal location (caecum to splenic flexure) (17); JNET type 2B or 3 pattern on magnifying NBI (13); and surface structural disruption, defined as visible fissuring, destroyed grooves, amorphous or irregular microvascular architecture, or focal loss of micro-pattern under white-light chromoendoscopy and/or NBI (18). Paris 0–II morphology was retained as a prespecified descriptive endoscopic variable and analysed separately because it was common in this chromoendoscopy-based cohort and was less specific as a stand-alone triage feature. The composite was designed as an interpretable bedside signal for exploratory risk enrichment rather than as a validated objective scoring system. The ≥2 threshold was chosen to reduce over-alerting from a single nonspecific feature while retaining clinical usability; descriptive sensitivity analyses using ≥1 and ≥3 cutoffs were performed to determine whether enrichment remained directionally consistent (**Supplementary Table 1**).

### 2.7 Statistical analysis

All analyses were performed using SPSS version 26.0 (IBM Corp., Armonk, NY, USA) and R version 4.2.2 (R Foundation for Statistical Computing, Vienna, Austria). Continuous variables (body mass index [BMI] and Boston Bowel Preparation Scale score) were assessed for normality using the Shapiro–Wilk test within each age group. Normally distributed continuous data were presented as mean ± standard deviation and compared across age strata using one-way ANOVA; *F* statistics are reported in **Table 1**. Non-normally distributed data were presented as median (interquartile range) and compared using the Kruskal–Wallis test, as appropriate. Categorical variables were expressed as *n* (%) and compared using the *χ²* test or Fisher’s exact test when expected cell counts were small, with *χ²* statistics reported for categorical comparisons.

**Table 1:** Baseline characteristics by age group and statistical tests.

Variable	36–45 years (n=58)	46–55 years (n=87)	56–65 years (n=97)	66–72 years (n=49)	χ²/F	P
Sex					0.85	0.548
Male, n (%)	28 (48.3)	46 (52.9)	54 (55.7)	29 (59.2)		
Female, n (%)	30 (51.7)	41 (47.1)	43 (44.3)	20 (40.8)		
BMI (kg/m²)	24.1 ± 2.8	25.0 ± 3.2	25.8 ± 3.5	26.3 ± 3.7	14.32	<0.001
H. pylori positivity, n (%)	17 (29.3)	32 (36.8)	43 (44.3)	25 (51.0)	10.21	0.018
Boston score (mean ± SD)	7.2 ± 1.1	7.5 ± 1.0	7.8 ± 0.9	8.0 ± 0.8	5.47	0.007

Data are presented as n (%) or mean ± SD, as appropriate. Comparisons across age groups were performed using the χ² test or Fisher’s exact test for categorical variables and one-way ANOVA for normally distributed continuous variables. Therefore, the statistic column reports χ² values for sex and H. pylori positivity, and F values for BMI and Boston score. BMI, body mass index; H. pylori, Helicobacter pylori.

Given the limited number of adenocarcinoma events, the primary outcome for regression modelling was prespecified as advanced colorectal neoplasia, defined as high-grade dysplasia (HGD) or adenocarcinoma, to improve statistical stability. Univariable logistic regression was first conducted to screen candidate predictors. Multivariable models were then constructed using clinically essential covariates to minimise overfitting, including age (per 10-year increment), sex, *H. pylori* status, lesion size ≥10 mm, proximal colon location and JNET type 2B/3 pattern. Adjusted odds ratios (ORs) with 95% confidence intervals (CIs) were reported.

For adenocarcinoma-only analyses, results were treated as exploratory due to sparse events, and penalised logistic regression with Firth correction was applied to reduce small-sample bias and separation. To avoid overfitting, adenocarcinoma-only regression was restricted to reduced Firth models using age-group increment and *H. pylori* status, while the red-flag composite was evaluated separately as a univariable enrichment signal because all adenocarcinoma cases were red-flag positive. Multiplicative interaction was assessed by including an age-group × *H. pylori* product term in the Firth model. Two-sided P < 0.05 was considered statistically significant. Data on smoking status, alcohol consumption, diet, physical activity, family history of CRC, key chemopreventive exposures (aspirin and other non-steroidal anti-inflammatory drugs [NSAIDs], statins, calcium and/or vitamin D supplementation) and prior *H. pylori* eradication history were not consistently captured in the retrospective endoscopy registry; therefore, these potential confounders could not be adjusted for, and residual confounding could not be excluded.

## 3 Results

### 3.1 Baseline characteristics

As shown in **Table 1**, a total of 291 participants who underwent colonoscopy screening and had histologically confirmed diagnoses were enrolled and stratified into 4 age groups: 36–45 years (*n* = 58), 46–55 years (*n* = 87), 56–65 years (*n* = 97), and 66–72 years (*n* = 49). Overall, 54.0% (157/291) of participants were men, with no significant difference in sex distribution across age groups (P = 0.548).

Mean BMI increased significantly with age (24.1 ± 2.8 kg/m² in the youngest group compared with 26.3 ± 3.7 kg/m² in the oldest; one-way ANOVA *F* = 14.32, P < 0.001). The prevalence of *H. pylori* positivity increased significantly with age, with the lowest rate in the 36–45-year group (17/58, 29.3%) and the highest in the 66–72-year group (25/49, 51.0%; *χ²* = 10.21, P = 0.018). Boston Bowel Preparation Scale scores also increased in an age-dependent manner (*F* = 5.47, P = 0.007).

### 3.2 Age-stratified lesion spectrum

As illustrated in **Table 2**, the within-cohort proportions of adenoma, SSL, HGD and adenocarcinoma all rose with advancing age. Adenocarcinoma prevalence increased from only 1 case (1.7%) in the 36–45-year group to 3 cases (6.1%) in the 66–72-year group. By contrast, the proportion of hyperplastic polyps declined markedly, from 58.6% to 14.3% (P for trend < 0.001).

**Table 2:** Distribution of lesion types in each age group.

Age group (year)	n	Adenoma n (%)	SSL n (%)	HGD n (%)	Adenocarcinoma n (%)	Hyperplastic polyp n (%)
36–45	58	20 (34.5)	2 (3.4)	1 (1.7)	1 (1.7)	34 (58.6)
46–55	87	37 (42.5)	5 (5.7)	4 (4.6)	1 (1.1)	40 (46.0)
56–65	97	50 (51.5)	8 (8.2)	7 (7.2)	3 (3.1)	29 (29.9)
66–72	49	28 (57.1)	5 (10.2)	6 (12.2)	3 (6.1)	7 (14.3)

Data are presented as n (%). Lesion classification was based on histopathological diagnosis according to the 2019 WHO Classification of Digestive System Tumours. SSL, sessile serrated lesion; HGD, high-grade dysplasia.

### 3.3 Endoscopic characteristics by age

**Table 3** shows that the proportions of proximal lesions, Paris 0–II morphology, JNET 2B/3 patterns, lesions ≥10 mm and surface structural disruption all increased with age. Specifically, proximal lesions increased from 27.6% to 48.9% (P = 0.009), lesions ≥10 mm increased from 10.3% to 34.7% (P < 0.001) and surface structural disruption increased from 6.9% to 28.6% (P < 0.001). Paris 0–II morphology showed a high baseline frequency and a weaker age trend, supporting its use as a descriptive feature rather than a component of the higher-specificity red-flag composite.

**Table 3:** Endoscopic characteristics by age group.

Characteristic	36–45 year group n=58	46–55 year group n=87	56–65 year group n=97	66–72 year group n=49	χ²	P
Proximal Colon Lesions	16 (27.6)	30 (34.5)	40 (41.2)	24 (48.9)	6.78	0.009
Paris 0–II	42 (72.4)	66 (75.9)	76 (78.4)	40 (81.6)	2.11	0.146
NBI JNET 2B/3	3 (5.2)	7 (8.0)	12 (12.4)	9 (18.4)	9.63	0.002
Lesions≥10 mm	6 (10.3)	14 (16.1)	24 (24.7)	17 (34.7)	15.41	<0.001
Surface structural disruption	4 (6.9)	10 (11.5)	18 (18.6)	14 (28.6)	13.25	<0.001

NBI, narrow-band imaging; JNET, Japan NBI Expert Team classification; HGD, high-grade dysplasia; ADC, adenocarcinoma; PPV, positive predictive value. Surface structural disruption was defined as visible fissuring, ulcer-like breakage, destroyed or interrupted pit/groove architecture, amorphous or irregular microvascular pattern, or focal spontaneous bleeding/erosion after exclusion of artefacts from biopsy, suction, or bowel preparation.

### 3.4 Age-related trends for adenocarcinoma and high-risk adenomas/high-grade dysplasia

**Table 4** summarises age-stratified regression results. Using the 36–45-year stratum as the reference, the odds of advanced neoplasia (HGD or adenocarcinoma) increased progressively with age, demonstrating a clear age gradient across the prespecified age strata. A similar, statistically significant age-dependent increase was observed for high-risk adenomas/HGD (P for trend < 0.001). Given the small number of adenocarcinoma events, adenocarcinoma-only age-stratified estimates were treated as exploratory and reported separately in the **Supplementary Materials** using Firth-penalised logistic regression for sparse outcomes (**Supplementary Table 2**).

**Table 4:** Participant-level age-stratified logistic regression results for advanced neoplasia and high-risk adenoma/HGD.

Age group (year)	Advanced neoplasia (HGD or adenocarcinoma) OR (95% CI)	High-risk adenoma/HGD OR (95% CI)	χ²	P
36–45	1.00 (Reference)	1.00 (Reference)	—	—
46–55	1.71 (0.32–9.11)	1.45 (1.02–2.07)	0.39	0.531
56–65	3.22 (0.68–15.24)	2.33 (1.55–3.51)	2.17	0.141
66–72	6.30 (1.29–30.74)	4.12 (2.11–8.05)	5.18	0.023

Advanced neoplasia was defined as HGD or adenocarcinoma and served as the primary outcome. Adenocarcinoma-only age-stratified estimates were treated as exploratory and are provided in **Supplementary Table 2**. This table reports participant-level age-stratified logistic regression estimates, with each participant classified according to the most advanced lesion.

### 3.5 Descriptive performance of the exploratory red-flag composite

The red-flag composite was defined as the presence of ≥2 of 4 higher-specificity endoscopic features in the most advanced lesion per participant: lesion size ≥10 mm, proximal location, JNET 2B/3 pattern or surface structural disruption. Paris 0–II morphology was analysed separately as a descriptive feature. As shown in **Table 5**, the positivity rate of the red-flag composite increased with age, from 3/58 (5.2%) in the 36–45-year group to 16/49 (32.7%) in the 66–72-year group. Descriptive sensitivity analyses showed that a ≥1-feature cutoff maximised capture but was less specific, whereas a ≥3-feature cutoff increased positive predictive value (PPV) at the cost of missing some adenocarcinoma events (**Supplementary Table 1**).

**Table 5:** Participant-level descriptive summary of the exploratory red-flag composite by age group.

Age group (year)	Red flag positive n (%)	Adenocarcinoma among red-flag positive (n/N)	PPV (%)	χ²	P
36–45	3 (5.2)	1/3	33.3	4.55	0.033
46–55	8 (9.2)	1/8	12.5		
56–65	18 (18.6)	3/18	16.7		
66–72	16 (32.7)	3/16	18.8		

PPV, positive predictive value. Red-flag positivity was defined as the presence of ≥2 of four higher-specificity endoscopic features: lesion size ≥10 mm, proximal colon location, JNET 2B/3 pattern, and surface structural disruption. Paris 0–II morphology was analysed separately as a descriptive feature and was not included in this composite. PPV estimates are descriptive and unstable because adenocarcinoma events were sparse. This table presents participant-level descriptive summaries rather than a predictive-model validation analysis.

The PPV of red-flag positivity for adenocarcinoma was 33.3% (1/3) in the 36–45-year group, 12.5% (1/8) in the 46–55-year group, 16.7% (3/18) in the 56–65-year group and 18.8% (3/16) in the 66–72-year group. Overall, red-flag positivity became more frequent with advancing age, and all adenocarcinoma cases occurred among red-flag-positive participants at the ≥2 threshold in this cohort. However, PPV did not increase monotonically by age; the relatively high PPV in the youngest stratum reflected the very small denominator (1 adenocarcinoma among only 3 red-flag-positive participants). These PPV estimates are descriptive and imprecise and should not be interpreted as externally validated predictive performance.

### 3.6 Independent predictors of advanced neoplasia and exploratory predictors of adenocarcinoma

In multivariable logistic regression with advanced neoplasia (HGD or adenocarcinoma) as the primary outcome, each 10-year increment in age, male sex, *H. pylori* positivity, lesion size ≥10 mm, proximal colon location and JNET 2B/3 pattern remained independently associated with higher odds of advanced neoplasia (**Table 6**).

**Table 6:** Participant-level multivariable logistic regression results for advanced neoplasia (HGD or adenocarcinoma).

Variable	OR (95% CI)	χ²	P
Age (per 10-year increase)	1.68 (1.31–2.16)	15.30	<0.001
Male sex	1.45 (1.02–2.07)	4.27	0.039
*H. pylori* infection (+)	1.72 (1.21–2.44)	8.75	0.003
Lesion size ≥10 mm	3.21 (2.11–4.89)	25.11	<0.001
Proximal colon location	1.89 (1.23–2.91)	8.06	0.004
JNET 2B/3 pattern	4.56 (2.67–7.79)	28.94	<0.001

The unit of analysis was the participant, with the most advanced lesion per participant used for classification. The primary outcome was advanced colorectal neoplasia, defined as high-grade dysplasia (HGD) or adenocarcinoma. Adjusted odds ratios (ORs) were estimated using multivariable logistic regression. Analyses restricted to adenocarcinoma alone were considered exploratory because of sparse events; therefore, Firth penalized logistic regression was applied and the corresponding results are provided in **Supplementary Table 3**. This table therefore presents participant-level multivariable logistic regression results.

Given the limited number of adenocarcinoma events, adenocarcinoma-only regression analyses were considered exploratory and were restricted to reduced Firth-penalised models to reduce small-sample bias, separation and overfitting; results are presented in the **Supplementary Materials** (**Supplementary Table 3**). In these exploratory analyses, age-group increment and *H. pylori* positivity showed positive but imprecisely estimated associations with adenocarcinoma, while red-flag positivity showed a strong enrichment signal but was evaluated only in a separate univariable Firth model because all adenocarcinoma events were red-flag positive.

### 3.7 Exploratory age–*Helicobacter pylori* effect modification (multiplicative interaction)

As shown in **Table 7**, adenocarcinoma prevalence increased numerically with age in both *H. pylori*-negative and *H. pylori*-positive participants, with the highest observed proportion among *H. pylori*-positive participants aged 66–72 years. To assess potential effect modification, an age × *H. pylori* product term was included in an exploratory Firth-penalised logistic model. Because there were only 8 adenocarcinoma events in total and several strata contained 0 or 1 event, confidence intervals were wide, and the multiplicative interaction was not statistically robust. These findings therefore provide only a hypothesis-generating signal, not reliable evidence of a clinically actionable age–*H. pylori* interaction.

**Table 7:** Participant-level exploratory Firth regression of adenocarcinoma by age group and H. pylori status.

Age group	H. pylori− n/N (%)	H. pylori+ n/N (%)	Firth OR (95% CI)*	Wald χ²	P
36–45	0/41 (0.0)	1/17 (5.9)	3.41 (0.12–219.8)	0.49	0.486
46–55	0/55 (0.0)	1/32 (3.1)	2.71 (0.10–165.4)	0.32	0.574
56–65	1/54 (1.9)	2/43 (4.7)	4.32 (0.38–75.2)	1.38	0.241
66–72	1/24 (4.2)	2/25 (8.0)	7.28 (0.60–135.6)	2.44	0.118

The two n/N columns show adenocarcinoma prevalence within each age–H. pylori stratum. ORs (95% CIs) were estimated using Firth penalised logistic regression and compare each H. pylori-positive age stratum with the H. pylori-negative 36–45-year reference stratum. Estimates are exploratory because only 8 adenocarcinoma events were available and several strata contained zero or one event. The multiplicative age × H. pylori interaction term was not statistically robust (interaction P = 0.684). The unit of analysis was the participant, and the table presents exploratory Firth logistic regression estimates.

## 4 Discussion

Based on a retrospective cohort of 291 participants who underwent screening colonoscopy and had histologically confirmed lesions, this study quantified age-associated gradients in colorectal lesion spectrum and high-risk endoscopic features across four prespecified age strata while treating *H. pylori* analyses as secondary and exploratory. From the 36–45-year to the 66–72-year group, the within-cohort proportion of adenocarcinoma increased from 1.7% to 6.1% and that of HGD increased from 1.7% to 12.2%. The positivity rate of the investigator-defined red-flag composite (≥2 of 4 higher-specificity features: lesion size ≥10 mm, proximal location, JNET 2B/3 pattern and surface structural disruption) rose from 5.2% to 32.7%. Its PPV for adenocarcinoma in the 66–72-year group was 18.8% (3/16), consistent with **Table 5**. Multivariable analysis showed that age (per 10-year increase), male sex, *H. pylori* positivity, lesion size ≥10 mm, proximal location and JNET 2B/3 pattern were associated with advanced neoplasia (HGD or adenocarcinoma). Adenocarcinoma-only and age–*H. pylori* interaction estimates were imprecise due to sparse events and should be regarded as hypothesis-generating.

Age is a consistent unmodifiable determinant of colorectal neoplasia, but its effect should be interpreted in the context of study population, screening indication, sex distribution and lesion definitions. In the present lesion-confirmed cohort, stratification across the four prespecified age groups showed a progressive increase in advanced neoplasia, with participants aged 66–72 having higher odds of HGD or adenocarcinoma than those aged 36–45 (OR 6.30, 95% CI 1.29–30.74). This estimate refers to advanced neoplasia, not adenocarcinoma alone. The finding is directionally consistent with prior colonoscopy studies showing that adenoma detection and the prevalence of advanced neoplasia increase with age (4–6). Mechanistically, ageing-associated DNA methylation drift, genomic instability, immune remodelling and chronic low-grade inflammation can facilitate the sequential acquisition of oncogenic alterations during colorectal tumourigenesis (7). These data support age-aware interpretation of lesion severity and endoscopic appearance, while prospective validation is required before such information can be incorporated into screening algorithms.

The red-flag composite was intended to translate routinely observed endoscopic features into a pragmatic enrichment signal rather than to create a validated risk score. Its components were selected because lesion size, proximal location, JNET type 2B/3 pattern on magnifying NBI and disrupted surface architecture are each linked to advanced histology, difficulty in optical diagnosis or concern for submucosal invasion in prior endoscopic literature (13, 16–18). In this cohort, the composite became more common with age and captured all observed adenocarcinoma cases at the ≥2 threshold, but the PPV varied across age strata and remained unstable because only 8 adenocarcinoma events were available. The sensitivity analysis suggested the expected trade-off: a cutoff of ≥1 feature was more inclusive but less specific, whereas a cutoff of ≥3 features was more specific but less sensitive. Therefore, the composite should be interpreted as an exploratory triage construct that may help identify lesions warranting careful optical assessment and planning for en bloc resection, referral or closer pathological correlation in future studies. It should not be considered an objective criterion or a substitute for established optical diagnosis systems. For example, in a 68-year-old patient with a 12-mm proximal lesion showing a JNET 2B pattern, three positive components could prompt the endoscopist to intensify optical assessment, document invasion-related features carefully and consider en bloc resection or referral to an advanced endoscopist, while definitive management remains guided by established classifications and histopathology.

Potential mechanisms underlying the association between *H. pylori* infection and colorectal neoplasia include systemic inflammation, gastrin-related endocrine effects, immune modulation and microbiome disturbance. In the present study, *H. pylori* positivity was associated with advanced neoplasia in the multivariable model, but this association should be interpreted cautiously because eradication history, strain virulence factors, gastric atrophy status and other behavioural or pharmacological risk modifiers were unavailable. The age–*H. pylori* analysis for adenocarcinoma was retained only to describe an observed pattern and to generate hypotheses for future studies with an adequate number of adenocarcinoma events.

This study has several limitations. First, its single-centre retrospective design and restriction to participants with histologically confirmed lesions may have introduced selection bias, as individuals with normal colonoscopy findings were not included. As a result, lesion proportions reflect the distribution within a lesion-confirmed screening cohort and may overestimate lesion prevalence compared with estimates from a full screening population. Second, the overall sample size and, in particular, the number of adenocarcinoma events were limited, which reduced the statistical power of the analyses and the stability of adenocarcinoma-only regression and interaction estimates. Third, smoking status, alcohol consumption, dietary pattern, physical activity, family history of CRC, key chemopreventive exposures (aspirin and other NSAIDs, statins, calcium and/or vitamin D supplementation), prior *H. pylori* eradication history and *H. pylori* virulence genes (e.g. *cagA* and *vacA*) were not consistently captured; therefore, residual confounding could not be excluded. Fourth, although the red-flag composite is pragmatic and easy to apply, it is investigator-defined, internally assessed and not externally validated; some components, especially surface structural disruption, remain subject to interobserver variability. Future prospective multicentre cohorts with complete risk-factor capture, standardised endoscopic image review and external validation are needed before the composite can be used as a clinical decision tool.

## 5 Conclusion

In summary, this study demonstrates a clear age-related gradient in colorectal lesion severity and prespecified endoscopic high-risk features in a lesion-confirmed screening colonoscopy cohort. The pragmatic red-flag composite may identify lesions enriched for advanced pathology, but it remains an exploratory, investigator-defined construct rather than a validated scoring system. Findings involving *H. pylori* status, including those from the age–*H. pylori* interaction analysis for adenocarcinoma, are hypothesis-generating because of sparse events and do not support changes to current screening algorithms. Prospective studies with larger numbers of adenocarcinoma events, comprehensive adjustment for relevant risk factors and external validation are warranted.

## Data Availability

The original contributions presented in the study are included in the article/**Supplementary Material**. Further inquiries can be directed to the corresponding authors.

## Supplementary material

Supplementary Table 1 Prespecified red-flag composite: components, definitions, and threshold sensitivity.

Supplementary Table 2 Exploratory age-stratified adenocarcinoma-only estimates.

Supplementary Table 3 Exploratory adenocarcinoma-only regression analyses (Firth penalized logistic regression).
