# Supplementary material for: Age-stratified gradients of colorectal neoplasia and an exploratory endoscopic red-flag composite in a lesion-confirmed screening colonoscopy cohort
Source: Front Oncol. 2026 Oct 2;16:1842525. doi: 10.3389/fonc.2026.1842525 (PMC13672195; doi:10.3389/fonc.2026.1842525)
Supplement: Supplementary file 1 [file Table1.docx]

**Supplementary Table S1. Prespecified red-flag composite: components, definitions, and threshold sensitivity**

| **Item** | **Name** | **Definition/result** | **Rationale** |
| --- | --- | --- | --- |
| 1 | Size ≥10 mm | Largest diameter ≥10 mm | Larger lesions carry higher advanced-histology risk. |
| 2 | Proximal location | Cecum to splenic flexure | Linked to higher-risk or missed lesions. |
| 3 | JNET 2B/3 | Magnifying NBI type 2B or 3 | Suggests HGD and/or invasive cancer. |
| 4 | Surface disruption | Fissures, destroyed grooves, irregular vessels, or loss of micro-pattern | May indicate advanced histology or invasion. |
| ≥1 | Sensitivity cutoff | 156/291 positive; ADC 8/156; PPV 5.1% | Highest capture but low specificity. |
| ≥2 | Main cutoff | 45/291 positive; ADC 8/45; PPV 17.8% | Balanced capture and specificity. |
| ≥3 | Sensitivity cutoff | 19/291 positive; ADC 5/19; PPV 26.3% | Higher PPV but lower sensitivity. |

Composite definition: Red-flag positivity was defined as ≥2 of the four higher-specificity features above, assessed using the most advanced lesion per participant. Paris 0–II morphology was analysed separately as a descriptive endoscopic feature.

Sensitivity analyses: Alternative cutoffs of ≥1 and ≥3 were evaluated descriptively. The ≥1 cutoff maximised capture but had low PPV, whereas the ≥3 cutoff increased PPV but missed some adenocarcinoma events; therefore, the ≥2 threshold was retained for the main descriptive analysis.

Note: NBI, narrow-band imaging; JNET, Japan NBI Expert Team classification; PPV, positive predictive value.

Supplementary Table S2. Exploratory age-stratified adenocarcinoma-only estimates

| Age group (n) | ADC n (%) | Firth OR (95% CI) | χ² | P |
| --- | --- | --- | --- | --- |
| 36–45 (58) | 1 (1.7) | 1.00 (Reference) | — | — |
| 46–55 (87) | 1 (1.1) | 0.66 (0.07–6.66) | 0.12 | 0.728 |
| 56–65 (97) | 3 (3.1) | 1.42 (0.20–10.03) | 0.12 | 0.725 |
| 66–72 (49) | 3 (6.1) | 2.89 (0.40–20.67) | 1.11 | 0.292 |

Note: ADC, adenocarcinoma. Adenocarcinoma-only estimates were derived using Firth penalised logistic regression with the 36–45-year group as the reference. These estimates are exploratory because only eight adenocarcinoma events were observed; wide confidence intervals indicate substantial imprecision.

Supplementary Table S3. Exploratory adenocarcinoma-only regression analyses (Firth penalized logistic regression)

| Variable | Firth OR (95% CI) | χ² | P |
| --- | --- | --- | --- |
| Reduced model: age + H. pylori |  |  |  |
| Age increment | 1.57 (0.77–3.22) | 1.53 | 0.216 |
| H. pylori positive | 3.52 (0.82–15.10) | 2.86 | 0.091 |
| Univariable model: red-flag |  |  |  |
| Red-flag positive | 111.75 (6.27–1991.51) | 10.30 | 0.001 |

Note: Estimates are exploratory because only eight adenocarcinoma events were observed. The reduced Firth model included age as a 10-year age-group increment and H. pylori status to avoid overfitting. The red-flag composite was evaluated separately as a univariable Firth model because all adenocarcinoma cases were red-flag positive, creating separation. Results should be interpreted as enrichment signals rather than validated prediction estimates. The primary outcome of the main analysis was advanced neoplasia (HGD or adenocarcinoma), reported in Table 6.
